# Asymmetry between Activators and Deactivators in Functional Protein Networks

**DOI:** 10.1038/s41598-020-66699-y

**Published:** 2020-06-23

**Authors:** Ammar Tareen, Ned S. Wingreen, Ranjan Mukhopadhyay

**Affiliations:** 1 Department of Physics, Clark University, Worcester, Massachusetts 01610 USA; 2 Lewis-Sigler Institute for Integrative Genomics, Carl Icahn Laboratory, Washington Road, Princeton, New Jersey 08544 USA; 3 Department of Molecular Biology, Princeton University, Princeton, NJ 08544 USA; 4 Present Address: Simons Center for Quantitative Biology, Cold Spring Harbor Laboratory, Cold Spring Harbor, New York 11724 USA

**Keywords:** Biological physics, Biochemical reaction networks

## Abstract

Are “turn-on” and “turn-off” functions in protein-protein interaction networks exact opposites of each other? To answer this question, we implement a minimal model for the evolution of functional protein-interaction networks using a sequence-based mutational algorithm, and apply the model to study neutral drift in networks that yield oscillatory dynamics. We study the roles of activators and deactivators, two core components of oscillatory protein interaction networks, and find a striking asymmetry in the roles of activating and deactivating proteins, where activating proteins tend to be synergistic and deactivating proteins tend to be competitive.

## Introduction

Biological oscillators are ubiquitous^1–3^ and they are often quite complex with many interacting components^4,5^. How has evolution arrived at such complex networks^6^ where oscillations arise from interactions among a large number of components? A reasonable hypothesis is that complex bio-oscillators evolved from simpler core oscillatory modules. While biological oscillators often involve both genetic and protein components, it has been suggested that in many systems the protein circuit acts as the core oscillator^7^. With this in mind, we focus here on oscillatory protein networks, where oscillations emerge from the interplay between positive and negative feedback loops. A central biologically motivated question is what are the evolutionary design principles of oscillatory protein networks, and how does complexity evolve in such systems?

The regulation of function in protein interaction networks is often achieved by post-translational modifications of component proteins. A common example is phosphorylation and dephosphorylation^8,9^. In most cases, an activating protein (e.g. a kinase) covalently modifies a target to activate it, and a deactivating protein (e.g. a phosphatase) reverses this change. However, despite this symmetry at the molecular level, at the network level activation and deactivation have distinct roles, and so it is natural to ask if there is an asymmetry in the way activators and deactivators are organized in protein networks. Recent studies have highlighted the asymmetry between auto-activation and auto-deactivation in oscillatory networks. In their computational work, Castillo-Hair *et al*.^10^, employing Michaelis-Menten kinetics and considering different network architectures, noted that auto-activation is responsible for producing the most robust oscillators. Additional works have explored the robustness of biological oscillators to network topology changes^11^ and found that auto-activation arises as an important and common motif for functions other than oscillations, such as multi-stability^12–14^. Moreover, in the context of protein network organization, Smoly *et al*.^15^ performed quantitative analyses of large-scale “omics” datasets from yeast, fly, plant, mouse, and humans and uncovered an asymmetric balance between kinases and phosphatases - each organism contained many different kinases, and these were balanced by a small set of highly abundant phosphatases. Motivated by this study, we ask whether an asymmetry in protein organization can arise from the intrinsically different roles that activators and deactivators play in protein-interaction networks.

## Evolutionary Model

To address this question, we adopt a physically-based protein-protein interaction model that allows us to map from sequence space to interactions and consequently to the oscillatory dynamics of enzymes, in order to study the evolution of oscillatory networks^16,17^. The model bridges multiple timescales, in particular, the short timescale of the dynamics of active enzyme concentrations and the much longer timescale of network evolution. As in the original network model^16^, we include cooperativity by assuming that activation or deactivation of a target (itself either an activator or a deactivator) requires $h$ independent binding/modification events, with partially modified intermediates being short lived. This yields the following chemical processes for the two classes of proteins,

1 $$\begin{array}{lll}h{A}_{i}^{\ast }+{A}_{j}/{D}_{j} & \mathop{\to }\limits^{{k}_{ij}} & h{A}_{i}^{\ast }+{A}_{j}^{\ast }/{D}_{j}^{\ast },\\ h{D}_{i}^{\ast }+{A}_{j}^{\ast }/{D}_{j}^{\ast } & \mathop{\to }\limits^{{k}_{ij}} & h{D}_{i}^{\ast }+{A}_{j}/{D}_{j},\end{array}$$

where $a$ represents activators, $D$ represents deactivators, and an asterisk indicates the active form. Note that in our model activators and deactivators act both as enzymes and as targets for the action of other enzymes. The corresponding chemical kinetic equations can be approximated as (see Supplementary Material for details):

2 $$\begin{array}{rcl}\frac{d[{A}_{j}^{\ast }]}{dt} & = & \mathop{\sum }\limits_{i=1}^{m}\,{k}_{ij}{[{A}_{i}^{\ast }]}^{h}[{A}_{j}]-\mathop{\sum }\limits_{i{\prime} =1}^{n}\,{k}_{i{\prime} j}{[{D}_{i{\prime} }^{\ast }]}^{h}[{A}_{j}^{\ast }]+\alpha [{A}_{j}]-\beta [{A}_{j}^{\ast }],\\ \frac{d[{D}_{j}^{\ast }]}{dt} & = & \mathop{\sum }\limits_{i=1}^{m}\,{k}_{ij}{[{A}_{i}^{\ast }]}^{h}[{D}_{j}]-\mathop{\sum }\limits_{i{\prime} =1}^{n}\,{k}_{i{\prime} j}{[{D}_{i{\prime} }^{\ast }]}^{h}[{D}_{j}^{\ast }]+\alpha [{D}_{j}]-\beta [{D}_{j}^{\ast }],\end{array}$$

where $m$ and $n$ are the number of distinct types of activators and deactivators respectively, $\alpha$ and $\beta$ represent background activation and deactivation rates respectively, and $h$ represents the degree of cooperativity. We assume that the total concentration of each species is constant, e.g. for activators, $[{A}_{j}]+[{A}_{j}^{\ast }]=[{A}_{j,\text{total}}]$.

**Figure 1:**
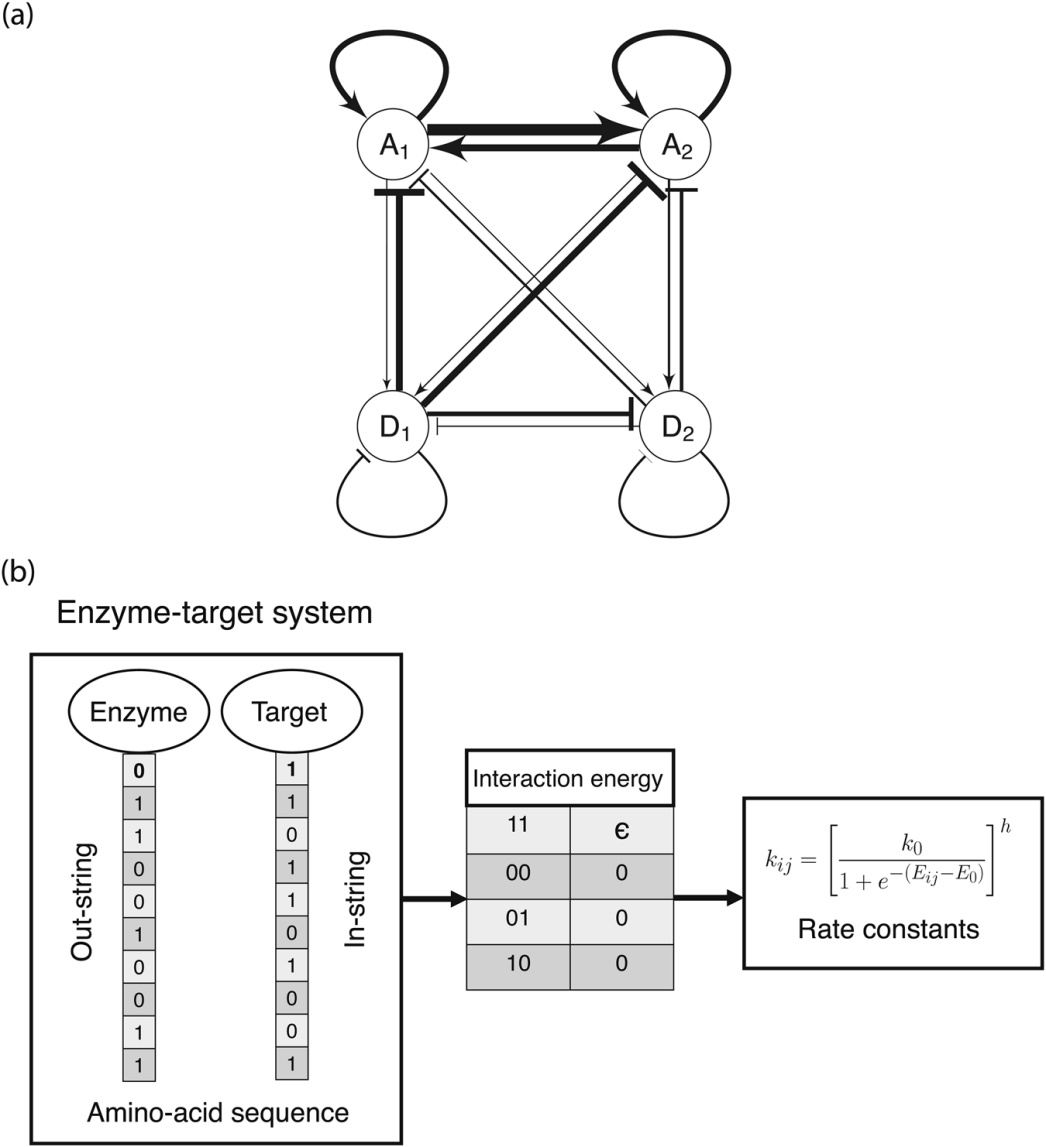
(**a**) Schematic representation of a 2-activator 2-deactivator protein interaction network. Nodes represent proteins while edges represent interactions with other proteins. This network is fully connected, i.e., every protein interacts with every other protein via activation or deactivation. Arrows represent activation while bars represent deactivation. Edge width is proportional to the strength of interaction. This network represents the state of a 2-activator 2-deactivator system after 10^4^ accepted mutations; this system was initially obtained by duplication of a 1-activator 1-deactivator network. (**b**) Amino-acid sequence to rate constant map depicting how 0s and 1s in the binary sequences interact to determine binding energies *E*_*ij*_. A 1:1 interaction produces an interaction energy equal to $\varepsilon$. All other interactions contribute zero interaction energy.

The chemical rate constants ${k}_{ij}$ are generally expected to be determined by protein-protein interaction strengths, which in turn are governed by amino-acid-residue interactions at specific molecular interfaces. As in Zulfikar *et al*.^16^, we assume protein interaction interfaces with the dominant contribution coming from hydrophobic interactions. For simplicity, as in previous work^16^, we associate a pair of interaction interfaces, an in-face and an out-face, with each protein, where a binary sequence, ${\overrightarrow{\sigma }}_{\text{in},\text{out}}$, of hydrophobic residues (1s) and hydrophilic residues (0s) are attributed to each interface. Interaction energy between a protein’s out-face (denoted by index $i$) and its target’s in-face (denoted by index $j$) is given by ${E}_{ij}=\varepsilon {\overrightarrow{\sigma }}_{\text{out}}^{i}\cdot {\overrightarrow{\sigma }}_{\text{in}}^{j}$, where $\varepsilon$ is the effective interaction energy between two hydrophobic residues and all energies are expressed in units of the thermal energy ${k}_{B}T$. The reaction rate is then given by:

3 $${k}_{ij}={\left[\frac{{k}_{0}}{1+\text{exp}[-({E}_{ij}-{E}_{0})]}\right]}^{h},$$

where ${E}_{0}$ indicates a threshold energy. Equation 3 represents two types of interactions, activation or deactivation, depending on the protein species involved, indexed by $\{i,j\}$. For example, ${k}_{{\text{A}}_{1},{\text{D}}_{1}}$ represents the reaction rate representing Activator-1 activating Deactivator-1, similarly ${k}_{{\text{D}}_{2},{\text{D}}_{1}}$ represents the reaction rate representing the Deactivator-2 deactivating Deactivator-1, and so on. Auto-activations and auto-deactivations are also allowed, e.g., ${k}_{{\text{A}}_{1},{\text{A}}_{1}}$, ${k}_{{\text{D}}_{2},{\text{D}}_{2}}$. Thus, our model represents a fully connected network architecture where every protein is allowed to interact with every other protein, including itself. This formulation provides a precise relationship between protein interface sequence (directly determined by the genome) and chemical rate constants. In the rate equations, $\alpha =\beta =1$ represent background activation and deactivation rates and set the unit of time. Other parameters are chosen to provide a large range for the rate constants ${k}_{ij}$ as a function of sequence and to keep the background rates small compared to the largest rate constant values. In our work, we set ${k}_{0}={10}^{4}$, $\varepsilon =0.2$, cooperativity $h=2$, ${E}_{0}=5$, sequence length representing an interface to be 25. Cooperativity is introduced to allow oscillations in relatively simple biomolecular networks. A schematic depicting this sequence-function relationship is shown in Fig. 1b.

For our evolutionary algorithm, we assume only point mutations, where in we replace a randomly chosen hydrophobic residue by a hydrophilic residue, or vice versa, at each evolutionary time step. Mutations are accepted if and only if they yield oscillatory network dynamics. Thus, our evolutionary scheme corresponds to assuming a population sufficiently small such that each new mutation is either fixed or entirely lost^18,19^ and represents a model of constrained neutral evolution (See Supplementary Material, section II, 10.1103/PhysRevE.97.040401).

## Activation and Deactivation Asymmetry

We start with a 1-activator 1-deactivator oscillatory network (the smallest network in our model that can generate oscillations) and duplicate it to generate a 2-activator 2-deactivator network (2A–2D), which is subsequently allowed to evolve^20^. Figure 1 shows a schematic of such a 4-node network after 10^4^ accepted mutations. The widths of the edges are proportional to the interaction strengths. As the network evolves, do certain interactions become stronger, fluctuate, or disappear, and why?

To answer these questions, we evolve the system over long evolutionary times (millions of accepted mutations), and consider the distributions of activation and deactivation asymmetries in rate constants, defined as.

4 $$\begin{array}{rcl}\text{Activation}\,\text{Asymmetry} & = & \frac{{k}_{{\text{A}}_{1},{\text{D}}_{1}}-{k}_{{\text{A}}_{2},{\text{D}}_{1}}}{{k}_{{\text{A}}_{1},{\text{D}}_{1}}+{k}_{{\text{A}}_{2},{\text{D}}_{1}}},\\ \text{Deactivation}\,\text{Asymmetry} & = & \frac{{k}_{{\text{D}}_{1},{\text{A}}_{1}}-{k}_{{\text{D}}_{2},{\text{A}}_{1}}}{{k}_{{\text{D}}_{1},{\text{A}}_{1}}+{k}_{{\text{D}}_{2},{\text{A}}_{1}}}.\end{array}$$

**Figure 2:**
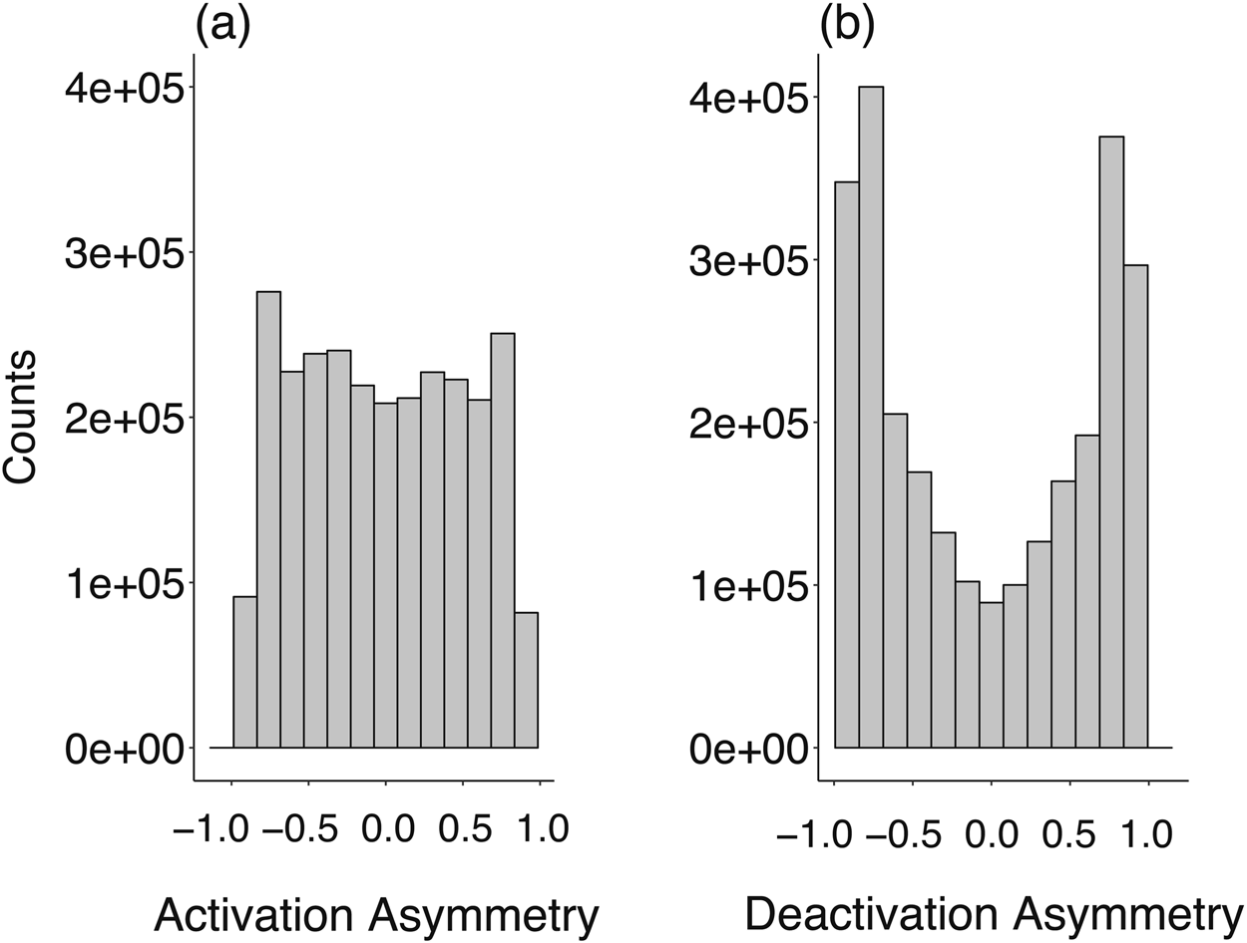
Distributions of (**a**) Activation Asymmetry and (**b**) Deactivation Asymmetry, constructed from 3 million accepted mutations for a 2-activator 2-deactivator network. Note that both distributions represent asymmetries in rate constants as defined in Eq. 4.

Activation Asymmetry is a measure of the difference in activation of a single deactivator by two activating proteins. Similarly, Deactivation Asymmetry measures the difference in deactivation of a single activator by two deactivating proteins. We expect these variables to be distributed differently if there is indeed an asymmetry present between activators and deactivators. Figure 2 shows distributions of Activation and Deactivation Asymmetries for a 2A–2D network, constructed from 2 million accepted mutations (We find that autocorrelation times of the rate constants are of the order of a few thousand accepted mutations; by evolving the system over millions of accepted mutations we ensure that we explore the space of rate constants sufficiently and not only over correlated samples, e.g. see Supplementary Fig. S1).

**Figure 3:**
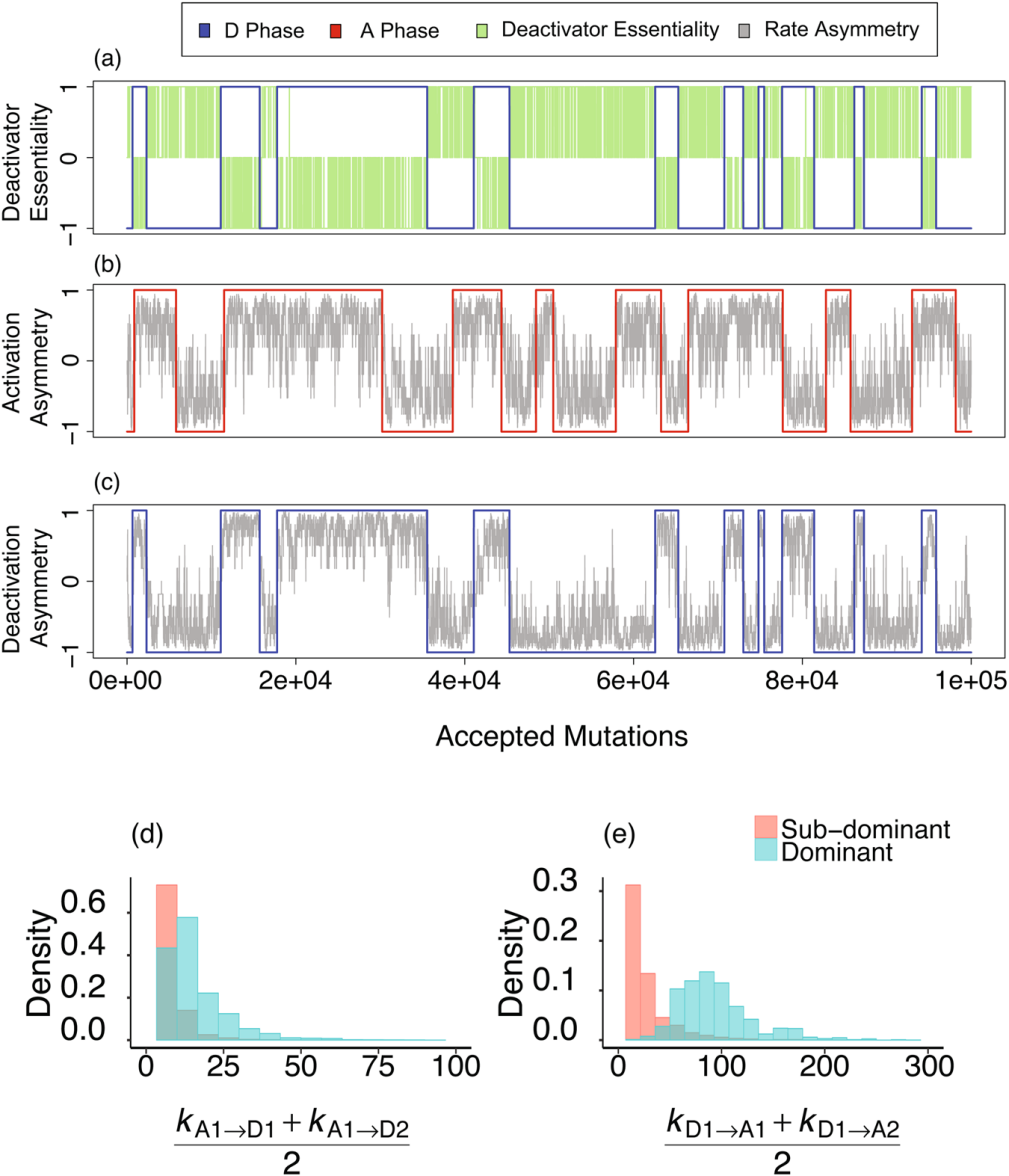
(**a**) Evolutionary traces of deactivator essentiality: on the *y*-axis, +1 indicates only Deactivator-1 is essential, −1 indicates only Deactivator-2 is essential, while 0 indicates both are essential (Since we find that states where both activators are individually inessential are very rare, approximately 0:001% of the total number of oscillatory states, we ignore such states for the purposes of the figure). We notice regions where Deactivator-1 remains essential and Deactivator-2 flip-flops in essentiality, which we designate as Deactivator-1 phase. In blue, +1 indicates Deactivator-1 phase and −1 indicates Deactivator-2 phase. (**b**) Activation Asymmetry and activator phases: in red, +1 indicates Activator-1 phase, −1 indicates Activator-2 phase, the line in gray shows Activation Asymmetry. (**c**) Deactivation Asymmetry: in blue, +1 indicates Deactivator-1 phase, −1 indicates Deactivator-2 phase, the line in gray shows Dectivation Asymmetry. Both Activation and Deactivation Asymmetry correlate strongly with their respective phases. (**d**) Distributions of average *k*_A,D_ for *A*_1_ dominant and subdominant. (**e**) Distributions of average *k*_D,A_ for *D*_1_ dominant and subdominant.

**Figure 4:**
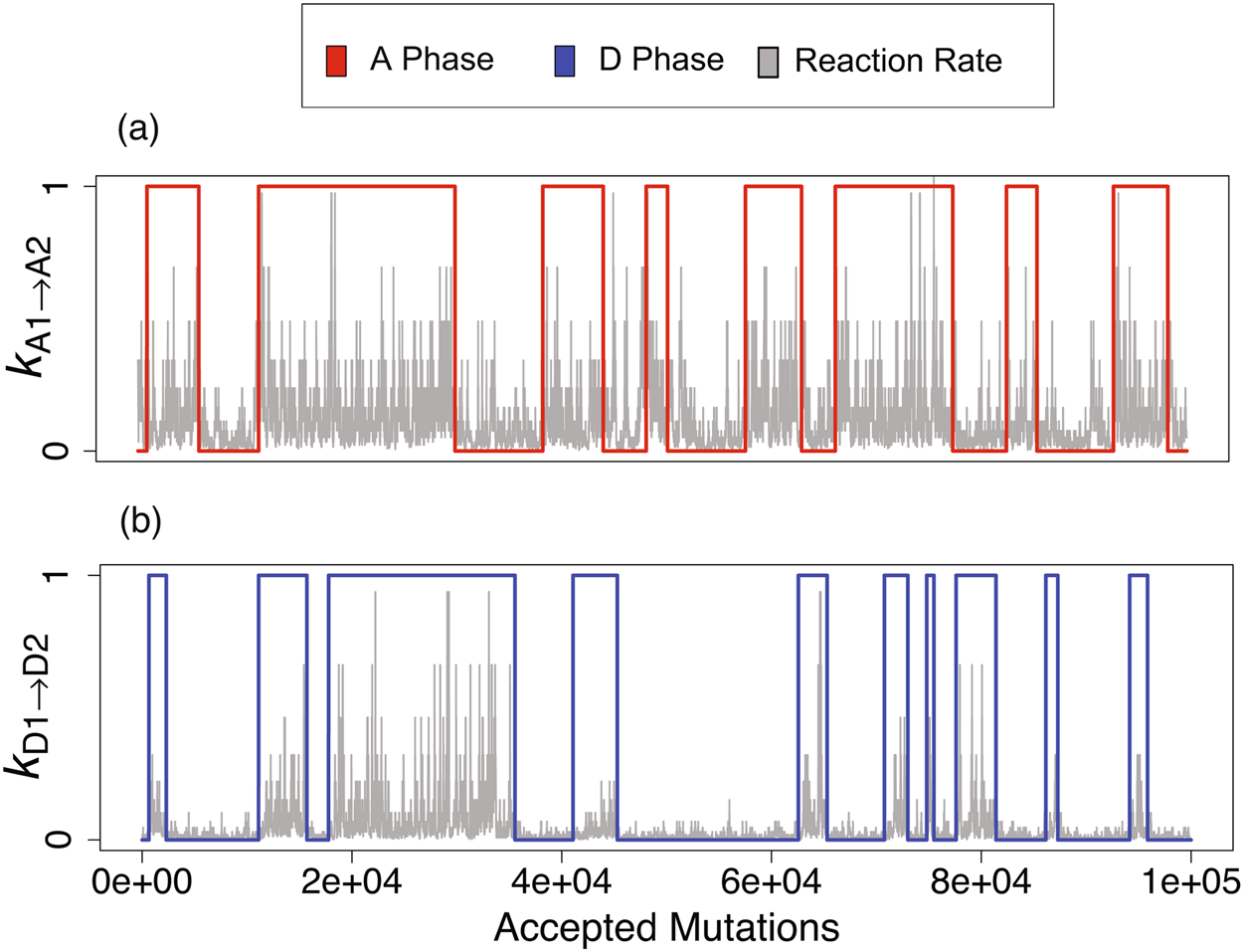
Synergistic activation between activators versus competitive deactivation between deactivators. The activator (deactivator) phase is shown in red (blue), while ${k}_{{\text{A}}_{1},{\text{A}}_{2}}$ $({k}_{{\text{D}}_{1},{\text{D}}_{2}})$ is shown in gray. +1 indicates Activator-1 (Deactivator-1) phase, while 0 indicates Activator-2 (Deactivator-2) phase. (**a**) The activation rate of an activator by the other activator, regardless of dominance or subdominance, does not correlate well with phase. (**b**) The deactivation rate of the subdominant deactivator is strongly correlated with the phase of the dominant deactivator.

**Figure 5:**
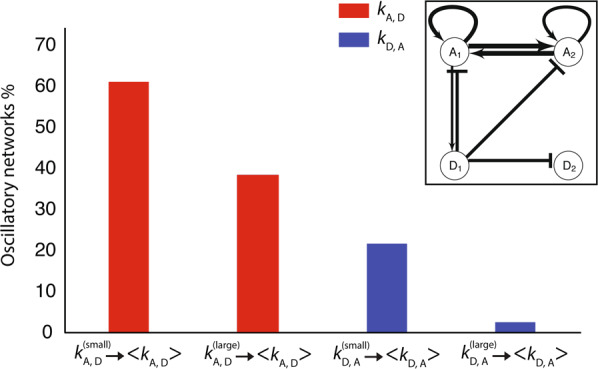
Comparison of networks that remain oscillating when ${k}_{\text{A},\text{D}}$ is replaced by $\langle {k}_{\text{A},\text{D}}\rangle$ (red) vs. the case when ${k}_{\text{D},\text{A}}$ is replaced by $\langle {k}_{\text{D},\text{A}}\rangle$ (blue) is shown. The y-axis represents the percentage after 1000 trials. Larger and smaller reaction rates were replaced by the average of both separately. The inset shows a schematic example of the typical network architecture that results after a large number of accepted mutations.

We indeed find a striking difference in the two distributions, with clear bimodality in the distribution of the Deactivation Asymmetry in contrast to the broad distribution in Activation Asymmetry. For activators, the difference between the two distributions signifies that the effect of both activators is comparable when acting on Deactivator-1. For deactivators acting on Activator-1, one of the two deactivators plays little to no role whereas the other deactivator deactivates dominantly. For example, if Deactivator-1 is deactivating Activator-1 with high strength, i.e. a higher rate constant, then the strength of deactivation of Deactivator-2 acting on Activator-1 is relatively much smaller. In the rest of this paper, we will develop the ideas required to understand the origin of this difference.

As a first step toward understanding the difference in these distributions, we examine the dynamics of evolution. To this end, it is helpful to introduce the concept of protein essentiality: we define a protein as being essential if its removal from the network causes oscillations to stop. As the network evolves, we observe periods of time when only Activator-1 is always essential, while Activator-2 flip-flops in essentiality, and periods where Activator-2 remains essential while Activator-1 flip-flops. The same is true for deactivators (see Fig. 3a). It was argued in^16^ that these long evolutionary periods reflect the division of sequence space into regions or phases separated by geometric bottlenecks (in sequence space). We define the “phase” associated with a protein in terms of time periods during which it always stays essential. Given a time when Activator-2 is inessential, we identify the system as being in Activator Phase 1. As the system evolves, at an evolutionary time step when Activator-1 first becomes inessential, we infer that the system has entered Activator Phase 2, and so on. Figure 3(b,c) show that time series plots of Activation and Deactivaction Asymmetry correlate well with the protein phases. Typically, the magnitude of a rate constant for a protein acting on a target is higher if the protein is in its associated phase. Since both activators and deactivators display similar transitions between their respective phases, why is there a difference in the asymmetry distribution between activators and deactivators?

To better understand the difference in asymmetry distributions for activators versus deactivators, we introduce the concepts of dominant and subdominant proteins: in Activator Phase 1, we say Activator-1 is the dominant protein while Activator-2, which flip-flops in essentiality, is subdominant, with a similar definition for deactivators. Is there a relationship between whether a deactivator is dominant or subdominant and the strength of its associated chemical rate constants ${k}_{\text{D},\text{A}}$? For instance, in Fig. 1a, Deactivator-1 is dominant and its associated rate constants for suppressing the activators, ${k}_{\text{D},\text{A}}$, are also significantly stronger than those for the subdominant Deactivator-2. The distributions of ${k}_{\text{A},\text{D}}$ and ${k}_{\text{D},\text{A}}$ in dominant and subdominant phases depicted in Fig. 3(d,e) provide support for the conjecture that the chemical rate constants, ${k}_{\text{A},\text{D}}$ or ${k}_{\text{D},\text{A}}$, associated with the subdominant activator or deactivator are suppressed in comparison to the dominant activator or deactivator. Notice however, that while the distributions for the dominant and subdominant activators differ only modestly, the distributions for the dominant versus subdominant deactivators are strikingly different.

Based on the results in Fig. 3(d,e), we can now understand the bimodality in Deactivation Asymmetry depicted in Fig. 2 in terms of the pronounced suppression of ${k}_{\text{D},\text{A}}$ for the subdominant deactivator. Notice that this suppression arises naturally from neutral drift without any direct evolutionary selection pressure. To gain an intuitive understanding of this suppression, consider the following. An oscillatory cycle begins with low levels of activators and deactivators. Due to self-activation, the concentrations of activators in their active state start to rise, with the dominant activator typically leading the subdominant activator. The rising levels of activators cause the active deactivator concentrations to rise as well, with the dominant deactivator typically leading the subdominant deactivator. As the active level of the dominant deactivator rises, it starts to suppress both activators, leading to the peak and subsequent drop in active activator concentrations. This dynamics, necessary for sustained oscillations, is highly sensitive to the rate constants ${k}_{\text{D},\text{A}}$. Our hypothesis is that it is easier to generate sustained oscillations if the deactivation of the activators is strongly coupled to the active level of the leading (dominant) deactivator and only weakly coupled to the lagging (subdominant) deactivator. To check this, we carried out the following test: we let a 2A–2D network evolve for 1000 accepted mutations and replaced ${k}_{{\text{D}}_{1},{\text{A}}_{1}}$ and ${k}_{{\text{D}}_{2},{\text{A}}_{1}}$ by their average value (and the same for A_2_) and determined if the network continued to oscillate upon making this change. These results were then compared to the case where we replaced ${k}_{{\text{A}}_{1},{\text{D}}_{1}}$ and ${k}_{{\text{A}}_{2},{\text{D}}_{1}}$ by their average value (and the same for D_2_). For the deactivators acting on activators, only $\mathrm{19.1 \% }$ of these average-value substitutions resulted in oscillations. On the other hand, for activators acting on deactivators, $\mathrm{65.6 \% }$ of the average-value substitutions resulted in oscillators. These results imply that our network is able to yield oscillations relatively easily when the rates ${k}_{{\text{A}}_{1},{\text{D}}_{1}}$ and ${k}_{{\text{A}}_{2},{\text{D}}_{1}}$ are comparable (similarly true for activation of D_2_), but has difficulty producing oscillations when the rates ${k}_{{\text{D}}_{1},{\text{A}}_{1}}$ and ${k}_{{\text{D}}_{2},{\text{A}}_{1}}$ are comparable (similarly true for deactivation of A_2_). However, this test reveals only part of the picture, as replacement by the average reduces the effect of the larger rate constant and increases the effect of the smaller. Is it one or both of these changes that matter?

We answered this question by carrying out the same test as before but this time replacing the larger and the smaller ${k}_{\text{D},\text{A}}$ by their average separately, and determining whether the network continued to oscillate. For activators acting on deactivators, replacing the smaller ${k}_{\text{A},\text{D}}$ by the average resulted in $\mathrm{61.0 \% }$ oscillators, and replacing the larger ${k}_{\text{A},\text{D}}$ by the average resulted in $\mathrm{38.4 \% }$ oscillators. For deactivators acting on activators, replacing the smaller ${k}_{\text{D},\text{A}}$ by the average resulted in $\mathrm{21.6 \% }$ oscillators, and replacing the larger ${k}_{\text{D},\text{A}}$ by the average resulted in only $\mathrm{2.50 \% }$ oscillators. These results imply that either lowering ${k}_{\text{D},\text{A}}$ for the dominant deactivator or increasing ${k}_{\text{D},\text{A}}$ for the subdominant deactivator results in far fewer oscillators, with the effect being particularly pronounced in the case of the weakening dominant deactivator. By contrast, changing ${k}_{\text{A},\text{D}}$ for either the dominant or subdominant activator has a less pronounced effect on oscillations. These results are summarized in Fig. 5.

### Synergy and competition

This brings us to a further distinction in the relationship between activators versus that between deactivators. Activator-1, for example, can activate Activator-2, which in turn further activates Activator-1, so that they act synergistically and effectively increase the degree of cooperativity for autoactivation. This is different for deactivators: Deactivator-1 suppresses Deactivator-2, and vice versa, so that deactivators act competitively, with the dominant deactivator suppressing the subdominant deactivator. This important distinction is consistent with our observation that activation of one activator by the other, regardless of its dominance or subdominance, does not correlate well with protein phase, implying synergistic activation of both activators for all evolutionary periods (e.g., see Fig. 4a). On the other hand, the deactivation of one deactivator by the other correlates strongly with deactivator phase; this behavior implies that when a deactivator becomes dominant the rate at which it *deactivates* the subdominant deactivator typically increases; an example of this behavior can be seen in Fig. 4b. This idea of synergistic versus competitive interactions is further borne out by the observation that the dominant activator has a higher autoactivation rate, whereas a subdominant deactivator has a higher auto-deactivation rate (see Fig. S2, (See Supplementary Material)). We can quantify this difference by carrying out another rate replacement test: we let a 2A–2D network evolve for 1000 accepted mutations but now replace the smaller of ${k}_{{\text{A}}_{1},{\text{A}}_{2}}$ and ${k}_{{\text{A}}_{2},{\text{A}}_{1}}$ by the larger value and determine if the network continued to oscillate. These results are then compared to replacing the smaller of ${k}_{{\text{D}}_{1},{\text{D}}_{2}}$ and ${k}_{{\text{D}}_{2},{\text{D}}_{1}}$. Based on the above analysis, we expect oscillations to be more likely to persist in the case of activators than deactivators, since activators are more likely to cooperate. Indeed, we find that 42.2% of tests resulted in oscillators for activator rate replacement while only 22.6% of the tests resulted in oscillators in the case of deactivator rate replacement.

## Asymmetry in Cooperativity

We have discussed that the subdominant activator serves to increase the rate and also the cooperativity of effective autoactivation of the dominant activator. Is there an asymmetry in the role of cooperativity in autoactivation versus auto-deactivation in the context of oscillations? To address this question, we study a 1A–1D network and perform a stability analysis about its fixed points. To determine stability of a steady state, one must know the eigenvalues of the Jacobian matrix **J** of the system evaluated at the steady state^21^. However, for the special case of the two-node network, it is sufficient just to know the trace and determinant of the Jacobian. If the determinant $\text{det}({\bf{J}}) > 0$ and the trace $\text{tr}({\bf{J}}) < 0$, then the steady state is stable and we do not expect limit-cycle oscillations. We will show that the trace becomes negative and the determinant becomes positive for any value of exponent of autoactivation that is less than a critical value. For a 1A–1D network, the dynamics of the active fractions of concentrations are given by:

5 $$\frac{d[{A}^{\ast }]}{dt}={k}_{\text{A},\text{A}}{[{A}^{\ast }]}^{a}[A]-{k}_{\text{D},\text{A}}{[{D}^{\ast }]}^{2}[{A}^{\ast }]+\alpha [A]-\beta [{A}^{\ast }],$$

6 $$\frac{d[{D}^{\ast }]}{dt}={k}_{\text{A},\text{D}}{[{A}^{\ast }]}^{2}[D]-{k}_{\text{D},\text{D}}{[{D}^{\ast }]}^{b}[{D}^{\ast }]+\alpha [D]-\beta [{D}^{\ast }],$$

where we have left the exponents of the autoactivation and auto-deactivation as variables $a$ and $b$ respectively. We denote the fixed points of the system by ${[{A}^{\ast }]}_{0}$ and ${[{D}^{\ast }]}_{0}$; they are obtained by setting $d[{A}^{\ast }]/dt$ and $d[{D}^{\ast }]/dt$ to zero in Eqs. 5 and 6 and solving for the active fractions of concentration. The Jacobian matrix of the system at the fixed point is

$$J=[\begin{array}{cc}{a}_{11} & {a}_{21}\\ {a}_{12} & {a}_{22}\end{array}]$$

with components defined as follows:

- $${a}_{11}=a{k}_{A,A}(1-{[{A}^{\ast }]}_{0}){{[{A}^{\ast }]}_{0}}^{(a-1)}-{k}_{\text{A},\text{A}}{{[{A}^{\ast }]}_{0}}^{a}-{k}_{\text{D},\text{A}}{{[{D}^{\ast }]}_{0}}^{2}-\alpha -\beta $$
- $${a}_{12}=-\,2{k}_{\text{D},\text{A}}{[{A}^{\ast }]}_{0}{[{D}^{\ast }]}_{0}$$, (<0)
- $${a}_{21}=2{k}_{\text{A},\text{D}}{[{A}^{\ast }]}_{0}{[D]}_{0}$$, (>0)
- $${a}_{22}=-\,{k}_{\text{A},\text{D}}{{[{A}^{\ast }]}_{0}}^{2}-(b+1){k}_{\text{D},\text{D}}{{[{D}^{\ast }]}_{0}}^{b}-\alpha -\beta $$, (<0)

Inspection shows that ${a}_{12}$ is always negative, ${a}_{21}$ is always positive or equal to 0, and ${a}_{22}$ is always negative. ${a}_{11}$ can be positive or negative depending on the value of $a$. If ${a}_{11}\le 0$, then $\text{det}({\bf{J}}) > 0$ and $\text{tr}({\bf{J}}) < 0$, implying the absence of oscillations. It is thus necessary that ${a}_{11} > 0$ for the system to oscillate. To determine the role of cooperativity for producing oscillations, we rewrite Eq. 5 as

7 $${k}_{\text{A},\text{A}}{{[{A}^{\ast }]}_{0}}^{a}(1-{[{A}^{\ast }]}_{0})={k}_{\text{D},\text{A}}{{[{D}^{\ast }]}_{0}}^{2}{[A]}_{0}^{\ast }+\alpha {[A]}_{0}-\beta {[{A}^{\ast }]}_{0}.$$

Dividing Eq. 7 by ${[{A}^{\ast }]}_{0}$ and plugging into the expression for ${a}_{11}$, we find

8 $${a}_{11}=-\,\frac{a\alpha }{{[{A}^{\ast }]}_{0}}-{k}_{\text{A},\text{A}}{{[{A}^{\ast }]}_{0}}^{a}+(a-1)({k}_{\text{D},\text{A}}{{[{D}^{\ast }]}_{0}}^{2})+(a-1)(\alpha +\beta ).$$

Note that at $a=1$, corresponding to the absence of cooperativity in autoactivation, we have

9 $${a}_{11}=-\,\frac{a\alpha }{{[{A}^{\ast }]}_{0}}-{k}_{\text{A},\text{A}}{{[{A}^{\ast }]}_{0}}^{a} < 0,$$

so that $\text{det}({\bf{J}}) > 0$. Consequently, there can be no oscillations for $a=1$. We find also that there is no such constraint imposed by cooperativity in auto-deactivation, e.g. we verified numerically that both ${a}_{11}$ and ${a}_{11}+{a}_{22}$ can be greater than zero for $b=1$. We further verified that the asymmetry noted in this paper arises independent of the choice of initial conditions, so long as the network remains oscillatory. This analysis highlights the asymmetry in the role of cooperativity in autoactivation versus auto-deactivation for producing oscillations.

## Conclusion

In this paper, we employed a sequence-based mutational algorithm to study the evolution of oscillatory protein networks. Beginning from a core module of an activating and deactivating protein that are subsequently duplicated, we found that deactivators possess a high degree of Deactivation Asymmetry while activators do not display any such Activation Asymmetry (see Fig. 2). We can understand this asymmetry by the synergistic roles of activating proteins and competitive roles of deactivating proteins: when an activator becomes subdominant, the dominant activator in the network works to increase the former’s activity. On the other hand, the dominant deactivator suppresses the subdominant deactivator. Finally, we showed that cooperativity is required only in autoactivation and not in auto-deactivation to generate oscillations.

Our theoretical results imply strong asymmetries in activator versus deactivator essentiality and function. We believe that more experimental work will further reveal the impact of such asymmetric behavior in protein-protein networks. Indeed, recent studies have already found these asymmetries experimentally^15^ and computationally^10^. Additional recent work has also found that DNA-copy-number asymmetry affects the ability of a genetic system of activators and deactivators to oscillate^22^. We note that deterministic models present only an approximation to the behavior of biophysical oscillators *in vivo*, and that the evolutionary dynamics of biochemical networks, such as those that we study in this paper, are affected by the presence of biochemical noise^23^, including intrinsic noise and stochastic gene expression^24^. In particular, the presence of noise generally weakens the robustness of oscillations^25,26^. Here, we chose a deterministic model so that we might focus on understanding the evolutionary dynamics of biochemical oscillators using a sequence-based mutational algorithm, founded on the idea that in real systems a single mutation might influence multiple reactions. We note that an extension of this study to include noise and stochasticity could be implemented as in Tareen *et al*.^17^.

An interesting future direction will be to explore these ideas quantitatively using a partial information decomposition^27^, or to evolve our oscillatory networks using biophysically realistic fitness landscapes^28^. Another future direction might also be to extend our model to systems other than oscillatory networks, such as signaling networks, to theoretically investigate asymmetries in the organization and dynamics of activators and deactivators. Finally, it would be interesting to see how the asymmetric roles of activators and deactivators extend to networks where the number of nodes is not fixed and could change with evolution.

## Supplementary information

Supplementary Information.
