# Supplementary material for: Asymmetry between Activators and Deactivators in Functional Protein Networks
Source: Sci Rep. 2020 Jun 23;10:10131. doi: 10.1038/s41598-020-66699-y (PMC7311538; doi:10.1038/s41598-020-66699-y)
Supplement: Supplementary file 1 — Supplementary Information. [file 41598_2020_66699_MOESM1_ESM.pdf]

# Supplementary Information for Asymmetry between Activators and Deactivators in Functional Protein Networks

**Ammar Tareen[1,+], Ned S. Wingreen[2,3,*], and Ranjan Mukhopadhyay[1,**]**

[1]Department of Physics, Clark University, Worcester, Massachusetts 01610
[2]Lewis-Sigler Institute for Integrative Genomics, Carl Icahn Laboratory, Washington Road, Princeton, New Jersey 08544
[3]Department of Molecular Biology, Princeton University, Princeton, NJ 08544, USA
[+]Present Address: Simons Center for Quantitative Biology, Cold Spring Harbor Laboratory, Cold Spring Harbor, New York, 11724, USA
[*]wingreen@princeton.edu
[**]ranjan@clarku.edu

## ABSTRACT

Supplementary information.

## 1 Supplementary Figures

## 2 Chemical rate equations for system of interacting protein species

We adopt this calculation of the derivation of the chemical rate equations from[1]. For simplicity, let us consider a two-component system consisting of one species of activators (e.g. kinases), denoted by letter A, and one species of deactivators (e.g. phosphatases), denoted by letter D, which can be in active or inactive states. Activators (in their active state) act only on inactive targets and deactivators (in their active state) act only on active targets. The chemical kinetic equations governing the system are given by

$$\mathrm{A}^* + \mathrm{T}_l \underset{k^r_{1l}}{\overset{k^f_{1l}}{\rightleftharpoons}} \mathrm{A}^*\mathrm{T}_l \xrightarrow{r_{1l}} \mathrm{A}^* + \mathrm{T}^*_l \tag{S1}$$

$$\mathrm{D}^* + \mathrm{T}^*_l \underset{\tilde{k}^r_{1l}}{\overset{\tilde{k}^f_{1l}}{\rightleftharpoons}} \mathrm{D}^*\mathrm{T}^*_l \xrightarrow{\tilde{r}_{1l}} \mathrm{D}^* + \mathrm{T}_l \tag{S2}$$

where A/A$^*$, D/D$^*$ and T/T$^*$ denote activators, deactivators, and targets respectively ( $^*$ denotes active state). The target molecules can be either activators or deactivators, and we will adopt the notation that for the two-component system, $\mathrm{T}_1$ corresponds to activator A and $\mathrm{T}_2$ corresponds to deactivator D. The rate of change of concentrations of the active fraction of the target $\mathrm{T}_1$, an activator, is given by

$$\frac{d[\mathrm{T}^*_1]}{dt} = \left(2r_{11} + k^r_{11}\right)[\mathrm{A}^*\mathrm{T}_1] - k^f_{11}[\mathrm{A}^*][\mathrm{T}_1] - \tilde{k}^f_{11}[\mathrm{D}^*][\mathrm{T}^*_1] + \tilde{k}^r_{11}[\mathrm{D}^*\mathrm{T}^*_1] \tag{S3}$$

and for the active fraction of target $\mathrm{T}_2$, a deactivator, is given by

$$\frac{d[\mathrm{T}^*_2]}{dt} = r_{12}[\mathrm{A}^*\mathrm{T}_2] + \left(\tilde{r}_{12} + \tilde{k}^r_{12}\right)[\mathrm{D}^*\mathrm{T}^*_2] - 2\tilde{k}^f_{12}[\mathrm{D}^*][\mathrm{T}^*_2] \tag{S4}$$

Note that in these equations, although $[\mathrm{A}^*] = [\mathrm{T}^*_1]$ and $[\mathrm{D}^*] = [\mathrm{T}^*_2]$, we have written them separately to distinguish between the roles of the proteins as enzymes or targets. For each intermediate complex, the rate of change of concentration is given by

$$\frac{d[\mathrm{A}^*\mathrm{T}_l]}{dt} = k^f_{1l}[\mathrm{A}^*][\mathrm{T}_l] - (k^r_{1l} + r_{1l})[\mathrm{A}^*\mathrm{T}_l] \tag{S5}$$

$$\frac{d[\mathrm{D}^*\mathrm{T}^*_l]}{dt} = \tilde{k}^f_{1l}[\mathrm{D}^*][\mathrm{T}^*_l] - (\tilde{k}^r_{1l} + \tilde{r}_{1l})[\mathrm{D}^*\mathrm{T}^*_l].. \tag{S6}$$

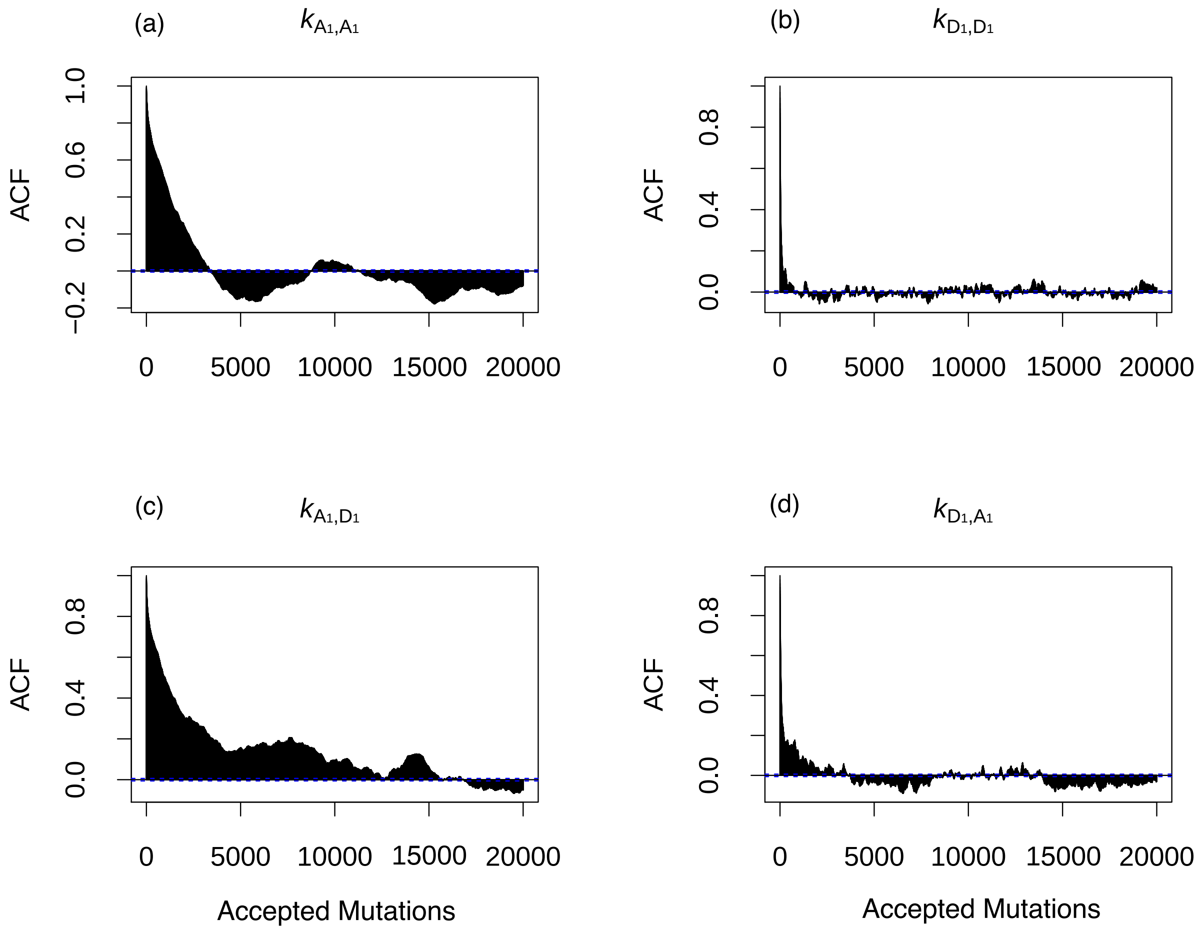


**Figure S1.** Autocorrelation plots of rate constants for (a) $k_{A_1,A_1}$, (b) $k_{D_1,D_1}$, (c) $k_{A_1,D_1}$, (d) $k_{D_1,D_1}$. 'ACF' stands for autocorrelation function. Autocorrelations between rate constants tend to 0 after a few thousand accepted mutations.

Under the assumption that the intermediate complex concentrations are at steady state (quasi-static approximation), we obtain

$$k^f_{1l}[\mathrm{A}^*][\mathrm{T}_l] - (k^r_{1l} + r_{1l})[\mathrm{A}^*\mathrm{T}_l] = 0$$

$$[\mathrm{A}^*\mathrm{T}_l] = \frac{k^f_{1l}}{k^r_{1l} + r_{1l}}[\mathrm{A}^*][\mathrm{T}_l] \tag{S7}$$

$$\tilde{k}^f_{1l}[\mathrm{D}^*][\mathrm{T}^*_l] - (\tilde{k}^r_{1l} + \tilde{r}_{1l})[\mathrm{D}^*\mathrm{T}^*_l] = 0$$

$$[\mathrm{D}^*\mathrm{T}^*_l] = \frac{\tilde{k}^f_{1l}}{(\tilde{k}^r_{1l} + \tilde{r}_{1l})}[\mathrm{D}^*_1][\mathrm{T}^*_l]. \tag{S8}$$

Substituting Eqs. (S7) and (S8) in Eq. (S3) yields

$$\frac{d[A^*]}{dt} = k_{\mathrm{A,A}}[A^*][A] - k_{\mathrm{D,A}}[D^*][A^*] \tag{S9}$$

where

$$k_{\mathrm{A,A}} = \frac{k^f_{11}}{1 + k^r_{11}/r_{11}},$$

$$k_{\mathrm{D,A}} = \frac{\tilde{k}^f_{11}}{1 + \tilde{k}^r_{11}/\tilde{r}_{11}}. \tag{S10}$$

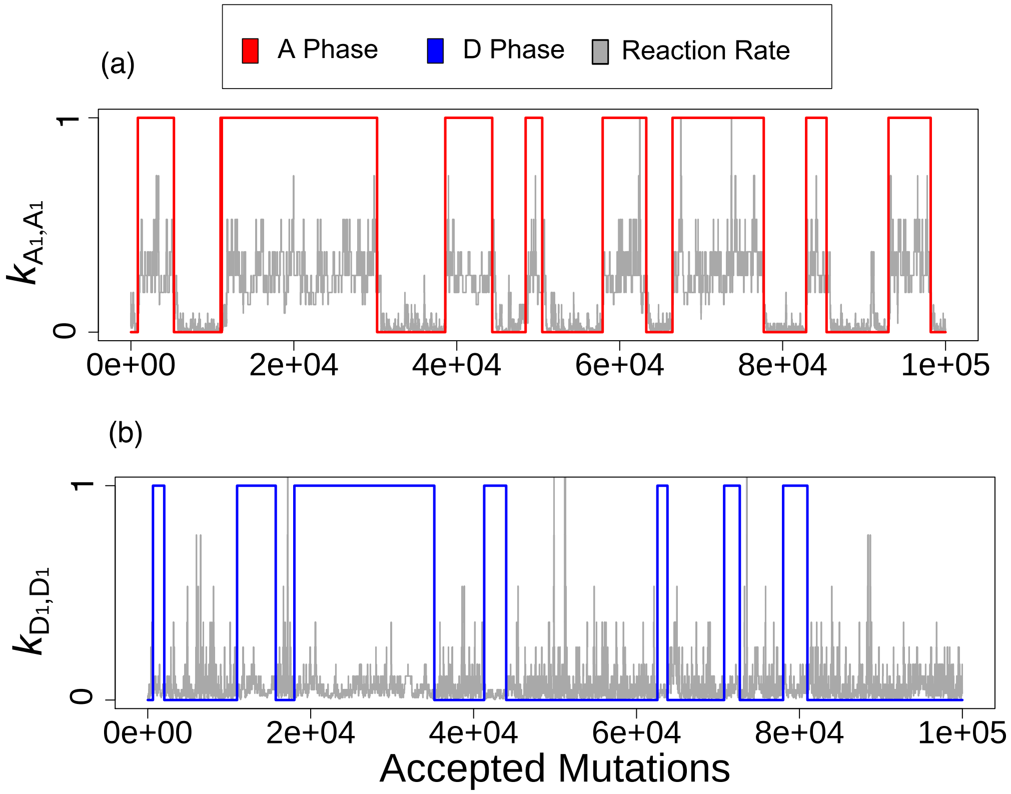


**Figure S2.** (a) Autoactivation and Activator phase. In red, 1 indicates Activator-1 phase and 0 indicates Activator-2 phase. (b) Auto-deactivation and Deactivator phase. In blue, 1 indicates Deactivator-1 phase and 0 indicates Deactivator-2 phase. The dominant activator has a higher autoactivation rate, whereas a subdominant deactivator has a higher auto-deactivation rate.

Similarly, we can obtain the second equation

$$\frac{d[D^*]}{dt} = k_{\mathrm{A,D}}[A^*][D] - k_{\mathrm{D,D}}[D^*]^2 \tag{11}$$

with similar relations for $k_{\mathrm{A,D}}$ and $k_{\mathrm{D,D}}$. Moreover, we assume a quasi-static approximation where ratios $k^f_{il}/(k^r_{il} + r_{il})$ and $\tilde{k}^f_{il}/(\tilde{k}^r_{il} + \tilde{r}_{il})$ are much smaller than one, corresponding to relatively short-lived intermediate complexes. Without loss of generality, we assume $k^f_{il}$ $(= \tilde{k}^f_{il})$, and $r_{il}$ $(= \tilde{r}_{il})$ are the same constants for all enzyme-target pairs, so that the only rate constants that depend on binding energies are $k^r_{il}$ and $\tilde{k}^r_{il}$. For these rates, we assume an Arrhenius-type form, e.g. $k^r_{il} = Ae^{-E_{il}/k_{\mathrm{B}}T}$ where $A$ is a constant and $E_{il}$ is the binding energy between enzyme represented by label $i$ and target represented by label $l$. If energy $E_{il}$ is measured in units of $k_{\mathrm{B}}T$ where $T$ is room temperature, we obtain

$$k_{ij} = k_0^{'} \left( \frac{1}{1 + e^{-(E_{ij} - E_0)}} \right), \tag{S12}$$

where $k_0^{'}$ and $E_0$ are constants, and the indices $i$ and $j$ stand for A or D. Similarly, $\tilde{k}_{jl} = k_0^{'}/(1 + e^{-(\tilde{E}_{jl} - E_0)})$. We have so far ignored background (enzyme-independent) activation and deactivation of target; we can incorporate this by adding a term of the form $\alpha[\mathrm{T}_l] - \beta[\mathrm{T}^*_l]$ to the right-hand side of Eqs. (S9) and (S11).

We next incorporate cooperativity within our minimal model. For simplicity of the discussion, we once again consider a two-component system (1 activator, 1 deactivator species), and assume target molecules can be in three states: inactive (T),

active ($T^*$), and partially phosphorylated ($T'$). The chemical kinetic equations governing the system are then of the form:

$$\begin{aligned} A^* + T &\xrightarrow{k'} A^* + T' \\ T' &\xrightarrow{k'_-} T \\ A^* + T' &\xrightarrow{k''} A^* + T^*. \end{aligned} \tag{S13}$$

Within the assumption of short-lived complexes, the rates of change of $T'$ and $T^*$ are given by

$$\begin{aligned} \frac{d[T']}{dt} &= k'[A^*][T] - k'_-[T'] - k''[A^*][T'] \\ \frac{d[T^*]}{dt} &= k''[A^*][T'] - \tilde{k}[D^*][T^*] + \alpha[T] - \beta[T^*]. \end{aligned} \tag{S14}$$

Applying the quasi-static approximation for $T'$ (valid for low concentrations of $[T']$), we obtain $[T'] = k'[A^*][T]/(k'_- + k''[A^*])$. If $k''[A^*] \ll k'_-$, we can further approximate $[T'] \approx k'[A^*][T]/k'_-$. We thus obtain

$$\frac{d[T^*]}{dt} = k[A^*]^2[T] - \tilde{k}[D^*][T^*] + \alpha[T] - \beta[T^*], \tag{S15}$$

where $k = k'k''/k_-$. Both $k'$ and $k''$ can be expected to be of the form in Eq. (S15), while $k_-$ as a spontaneous decay rate can be treated as a constant. For simplicity, we assume that the enzyme binding energies for both steps of phosphorylation are the same, and obtain

$$k = k_0 \left( \frac{1}{1 + e^{-(E-E_0)}} \right)^2, \tag{S16}$$

where $k_0$ is now a new constant. Cooperativity in deactivation is introduced along similar lines. We then generalize this to multiple activator/deactivator species, with the simplifying assumption that activators/deactivators involved in both stages belong to the same species, giving us Eqs. (2) in the main text.

## References


**1.** See Supplementary Material, section I [http://link.aps.org/supplemental/10.1103/PhysRevE.97.040401].
